# Synthesis, Characterization, and Catalytic Properties of Magnetic Fe_3_O_4_@FU: A Heterogeneous Nanostructured Mesoporous Bio-Based Catalyst for the Synthesis of Imidazole Derivatives

**DOI:** 10.3389/fchem.2020.596029

**Published:** 2020-12-01

**Authors:** Maryam Banazadeh, Sara Amirnejat, Shahrzad Javanshir

**Affiliations:** Heterocyclic Chemistry Research Laboratory, Chemistry Department, Iran University of Science and Technology, Tehran, Iran

**Keywords:** fucoidan, heterogeneous catalyst, imidazoles, superparamagnetic iron oxide nanoparticles, sulfated polysaccharides

## Abstract

In this protocol, Fucoidan (FU), a fucose-rich sulfated polysaccharide extracted from brown algae Fucus vesiculosus was used for *in situ* preparation of magnetic Fe_3_O_4_@FU. Nanoco magnetic properties of Fe_3_O_4_@FU were investigated by energy dispersive X-ray (EDX) spectroscopy, X-ray diffraction (XRD), Fourier transform infrared spectroscopy (FT-IR), field emission scanning electron microscopy (FESEM), transmission electron microscopy (TEM), Brunauer–Emmett–Teller (BET) adsorption method, and vibrating sample magnetometer (VSM). The catalytic activity of Fe_3_O_4_@FU was employed for the synthesis of tri- and tetra-substituted imidazoles through three- and four-component reactions respectively, between benzyl, aldehydes, NH_4_OAc and benzyl, aldehydes, NH_4_OAc, and amine under reflux in ethanol. It is worth nothing that excellent yields, short reaction times, chromatography-free purification, and environmental friendliness are highlighted features of this protocol.

## Introduction

Through extensive applications and potential in the chemical industry and preservation of the environment, the recently supported solid nanocatalysis has been faced with various attentions in catalysis science and technology (Amirnejat et al., 2013; Fereshteh and Shahrzad, 2020). To overcome the difficulty of catalyst separation, some magnetic heterogeneous catalysts with unique features and advanced functionalities suitable for a range of applications, including biological and environmental applications have been made (Pourian et al., 2018; Zaheri et al., 2018). The magnetic materials have gained more attention due to their combined physicochemical characteristics such as high surface area, high thermal and chemical stability, excellent biocompatibility and biodegradability, and efficient super magnetic behavior (Dolatkhah et al., 2018; Piri et al., 2019).

Natural biopolymers as an effective tool have given rise to a new method of producing degradable materials. Meanwhile, marine polysaccharides exhibit a vast variety of structures and could be considered as a novel natural source (Dekamin et al., 2016; Alipour et al., 2018; Dolatkhah et al., 2019). Nanomaterials based on marine polysaccharides have been considered as one of the most important topics of research in recent years, especially in chemical and bio-based research, due to biocompatibility and biodegradability, cheapness, non-toxicity, and abundance (Hemmati et al., 2016; Amirnejat et al., 2020a,b,c). Fucoidan refers to a type of polysaccharide which contains substantial percentages of L-fucose and sulfated ester groups, mainly derived from brown seaweed which has been extensively studied due to its numerous interesting biological activities (Gomez-Zavaglia et al., 2019; Zayed and Ulber, 2019, 2020).

**Scheme 1:**
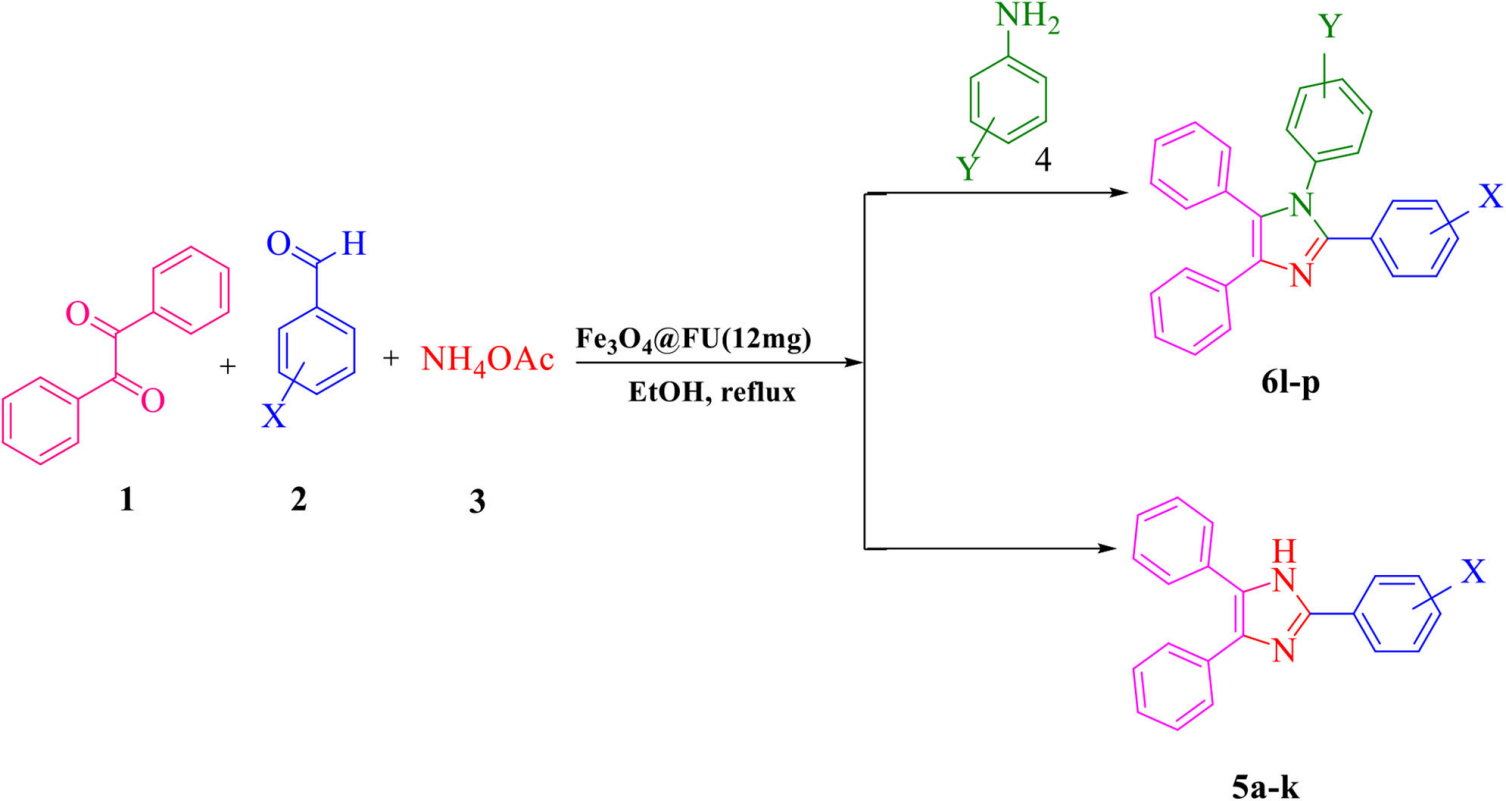
The synthesis of 2,4,5-triaryl-1*H*-imidazoles **5a-k** and 1,2,4,5- tetraaryl-1*H*-imidazoles **6l-p** *via* catalytic application of Fe_3_O_4_@FU.

In recent decades, it has been extensively represented that multi-component reaction (MCR) is an ideal tool for creating molecular diversity and complexity (Graebin et al., 2019). Meanwhile imidazoles, polar in nature and with a five-member ring structure, are one of the most important compounds showing a wealthy source of biologically important features such as inhibitors, fungicides, herbicides plant, anti-inflammatory, anticancer, antimicrobial, analgesic, and anti-tubercular activity (Shalini et al., 2010; Varzi and Maleki, 2019). Numerous approaches have been developed for the synthesis of 1,2,4,5-tetrasubstituted imidazoles, which can be prepared by a four-component cyclo condensation consisting of aldehyde, benzil, a primary amine and ammonium acetate in the presence of different catalysts such as BF_3_·SiO_2_ (Sadeghi et al., 2008), and silica gel/NaHSO_4_ (Karimi et al., 2006), while other main components are achieved by synthesis of tri-substituted imidazoles by the condensation of benzil derivatives, aryl aldehydes, and ammonium acetate catalyzed by different catalysts such as ZrCl_4_ (Shitole et al., 2015), sulfanilic acid (Gadekar et al., 2009), and chitosan (Zheng et al., 2019). However, some of these methodologies have some drawbacks, such as low yields, long reaction times, severe reaction conditions, and work up procedure. Herein, we report the *in situ* synthesis of a novel eco-friendly magnetic heterogeneous catalyst Fe_3_O_4_@FU for the synthesis of tri- and tetra-substituted imidazoles under reflux condition in ethanol (Scheme 1). Easy work up and separation, high product yields and short reaction times made this method effective and advantageous.

## Experimental

### Materials and Methods

All solvents and chemicals were purchased from Merck and Aldrich. All reactions and the purity of the products were monitored by thin-layer chromatography (TLC) using aluminum plates coated with silica gel F254 plates (Merck) using ethyl acetate and n-hexane as eluents. UV light with a wavelength of 254 nm was used for the detection of products. By using an Electro thermal 9100, melting points were determined in open capillaries. IR spectra were run on a 400s Shimadzu FTIR Spectrophotometer (as KBr pellets). ^1^H and ^13^C NMR spectra were recorded on a 500 MHz Bruker Avance DRX Spectrometer instrument using TMS as an internal standard and CDCl_3_, DMSO-*d6* as a solvent. The XRD patterns were obtained on an X-ray diffractometer (Holland, Philips Xpert, Co K, radiation, λ = 0.178897 nm). A Field Emission Scanning Electron Microscope (FE-SEM) with 15 KV, Mira3, Tescan), Thermal Gravimetric Analysis (TGA D-32609 from Hullhorst), and Transmission electron microscope (TEM, Philips –CM120, 100 KV) were used. An ultrasonic probe watt ultrasonic homogenizer 400 from Topsonics Co was used in room temperature for optimization of the reaction.

### Preparations of Fucoidan Powder

2.5 gr of algae Fucus vesiculosus was finely ground by a ball mill for 5 min and was placed in a round bottom 200 ml flask containing 100 ml of 96% ethanol, and was stirred for 12 h. Then, the suspension was centrifuged at 4,000 rpm for 15 min and the resulting powder was dried at 50°C for 1 h.

### Synthesis of Magnetic Fe_3_O_4_@FU Nanocomposite

**Scheme 2:**
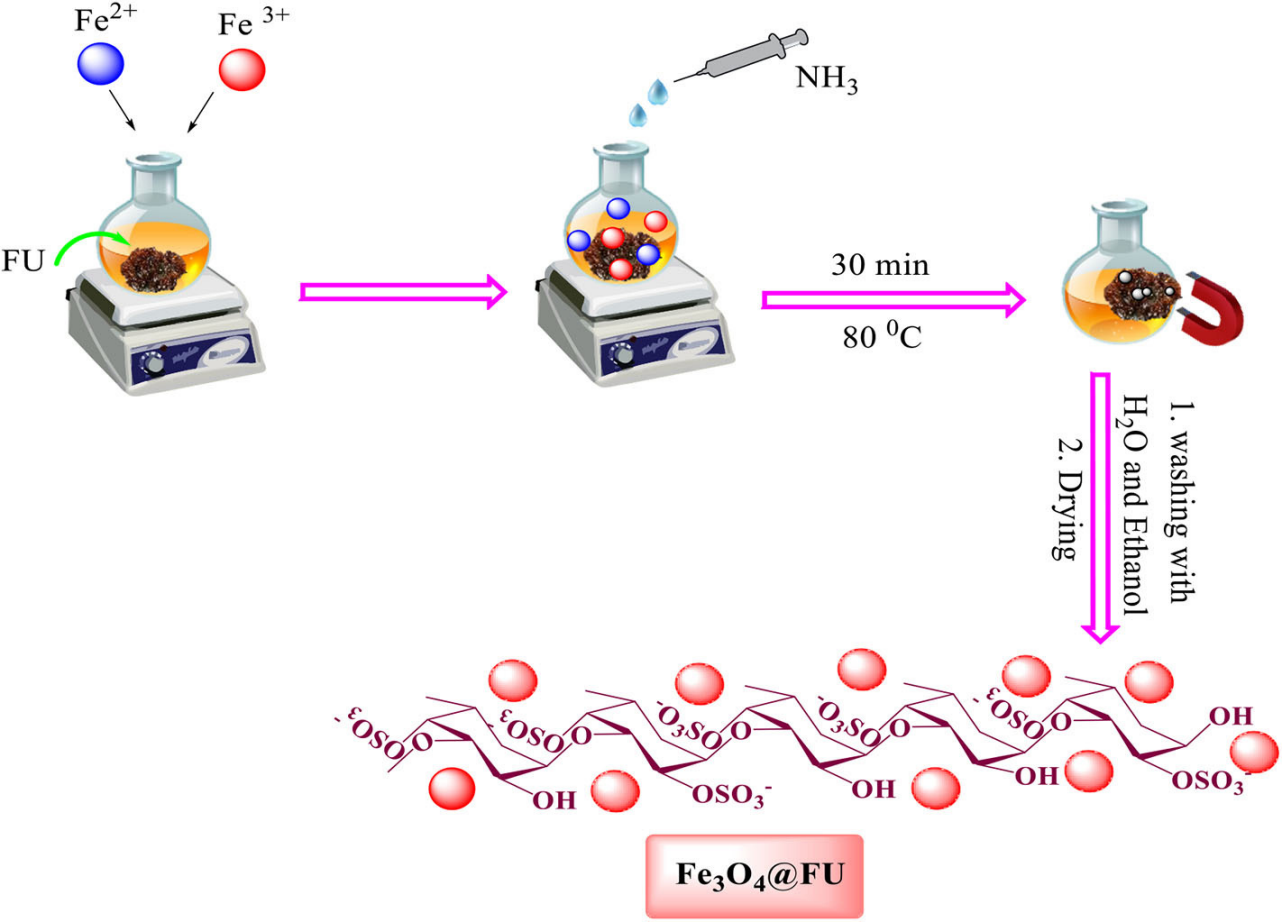
Preparation of Fe_3_O_4_@FU magnetic nanocomposite.

For the preparation of the Fe_3_O_4_@FU, 0.2 g of fucoidan powder, FeCl_2_.4H_2_O (2 g, 0.01 mol) and (5.5 g, 0.02 mol) of FeCl_3_ were used. 6H_2_O was dissolved in 100 ml of distilled water. Then the mixture was vigorously stirred under a nitrogen atmosphere at 80°C for 15 min to reach a uniform solution. The pH of the solution was then adjusted to 12 by the dropwise addition of an aqueous ammonia solution (25%). The mixture was stirred at 80°C for 45 min. The prepared magnetic nanoparticles were separated by an external magnet, finally washed with ethanol and DI, and dried for 6 h at 60°C (Scheme 2).

#### General Procedure for the Preparation of 2, 4, 5-Trisubstituted Imidazoles Derivatives

A mixture of benzil (210 mg, 1 mmol), aldehydes (1 mmol), NH_4_OAc (154 mg, 2 mmol), and Fe_3_O_4_@FU NPs (12 mg) in 3 ml EtOH was stirred under reflux conditions for the appropriate times. The progress of the reactions was monitored by TLC (eluent: EtOAc / n-hexane, 1: 3). After completion of the reaction, the catalyst was easily separated by an external magnet and then reused as such for the following experiment after being washed with EtOH and dried. The pure products were obtained by recrystallization from hot EtOH and then dried at 60°C for 1 h.

#### General Procedure for the Preparation of 1, 2, 4, 5-Tetrasubstituted Imidazoles Derivatives

Benzil (210 mg, 1 mmol), aldehydes (1 mmol), NH_4_OAc (77 mg, 1 mmol), amine (1 mmol), and Fe_3_O_4_@FU NPs (12 mg) in ethanol (3 mL) was stirred under reflux conditions until completion of the reaction which was monitored by TLC (eluent: EtOAc/n-hexane, 1:3). The catalyst was separated from the mixture by an external magnet, washed and dried for use in the next run. The crude products were filtered and recrystallized from ethanol.

## Results and Discussion

**Figure 1:**
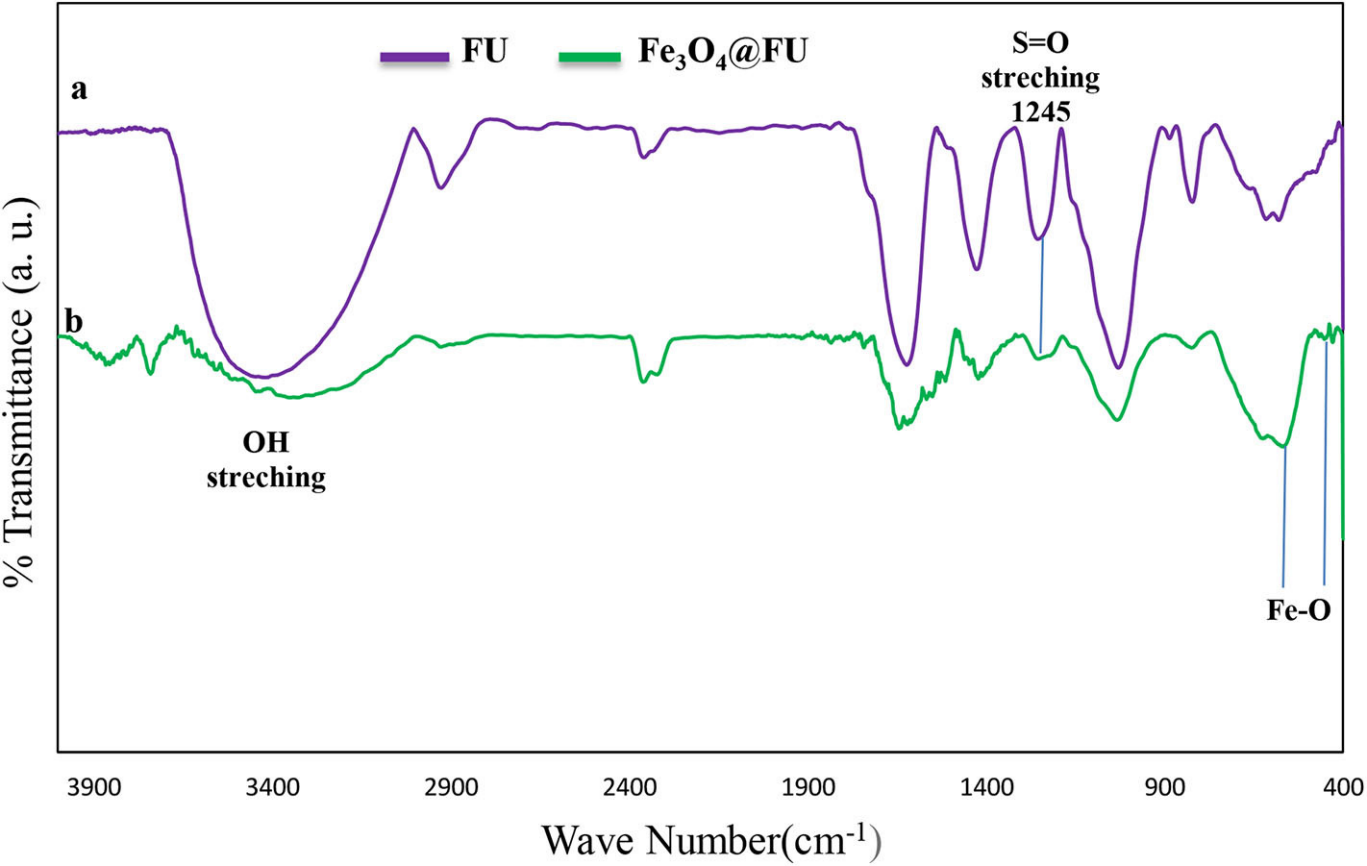
The FT-IR spectra for **(a)** FU, **(b)** Fe_3_O_4_@FU NPs.

Fe_3_O_4_@FU magnetic nanoparticles as a heterogeneous catalyst were characterized by Fourier transform infrared (FT-IR) spectral analysis. One strong broad band at 3,500 cm^−1^ was attributed to the stretching vibration due to the O-H of fucoidan and water. The appearance of the peak at 1,624 cm^−1^, attributed to significant polysaccharide chains (Figure 1a), is stronger than magnetic fucoidan (Figure 1b). The absorption band at 1,029 cm^−1^ indicated hemiacetal vibration at alcohol and ether functional groups in fucoidan structure. Furthermore, the peak at 1,240–1,255 cm^−1^ was related to the stretching vibration of S=O from the SO_3_H group. The presence of the metal oxide peaks of 570 and 455 cm^−1^ also exhibited in FT-IR of Fe_3_O_4_@FU acknowledged that the chemical structure of the magnetic nanoparticles have been preserved after the functionalization.

**Figure 2:**
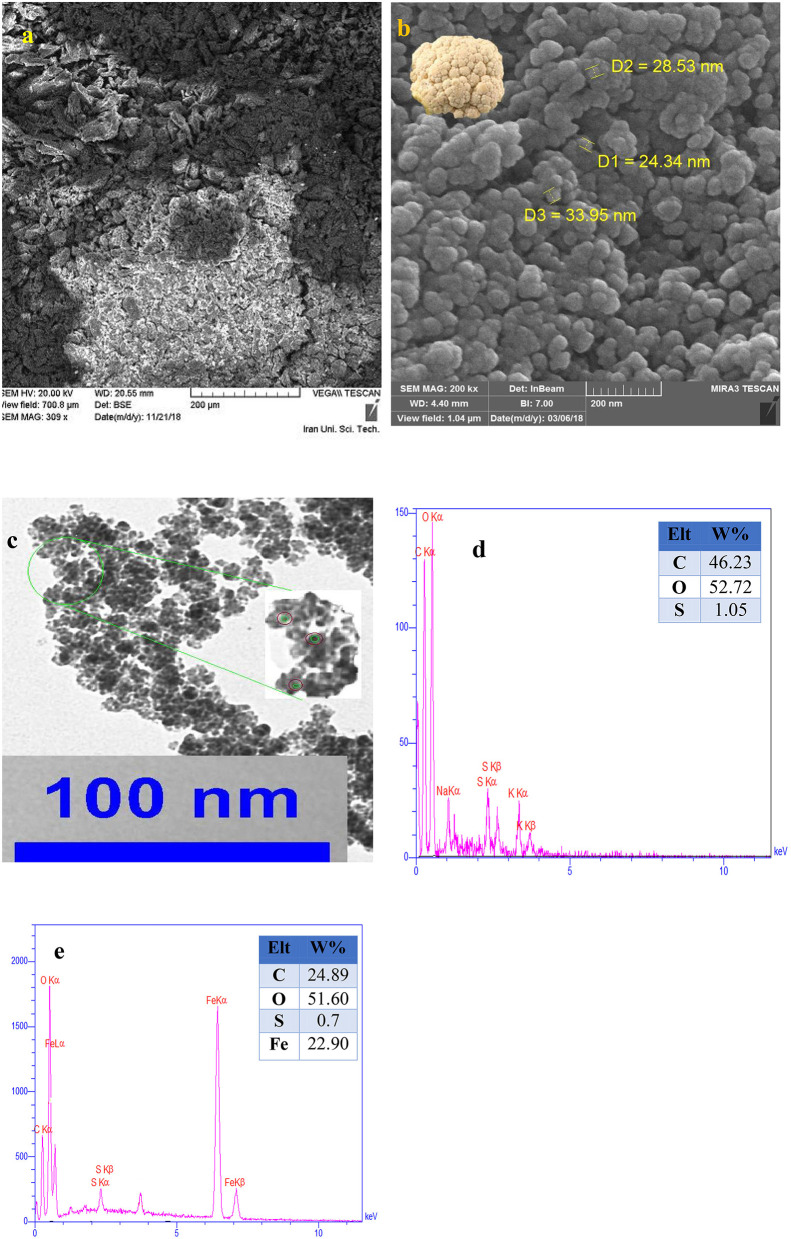
SEM images of **(a)** FU, **(b)** Fe_3_O_4_@FU, and **(c)** TEM images of Fe_3_O_4_@FU NPs. EDX analysis of **(d)** FU, and **(e)** Fe_3_O_4_@FU NPs.

Primarily, the size, structure, and morphology of FU, and the as prepared nanocomposite were investigated by SEM analyses (Figures 2a,b). As can be seen, Fe_3_O_4_@FU nanocomposites have a cauliflower-shaped morphology in which the average size distribution was around 24–33 nm. TEM analysis of the as-synthesized Fe_3_O_4_@FU showed that the Fe_3_O_4_@FU NPs have a core–shell structure (Figure 2c). Simultaneously, the elemental composition of Fe_3_O_4_@FU and FU were studied by EDX analysis (Figures 2d,e) which confirmed the presence of O, C, Fe, and S elements constituted in the nanocomposite.

**Figure 3:**
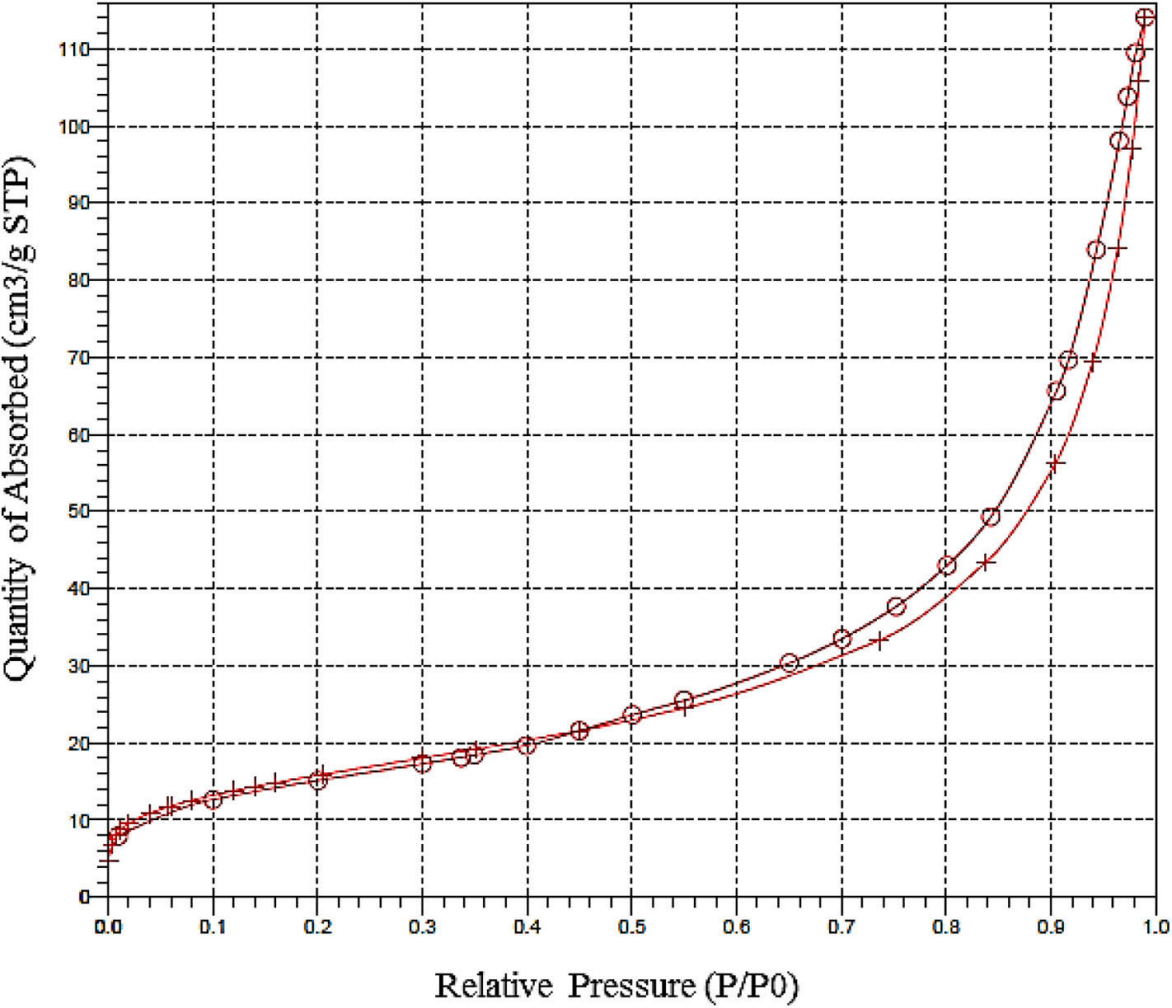
BET curve of Fe_3_O_4_@FU NPs.

The surface area and pore size distributions of the Fe_3_O_4_@FU were analyzed by N_2_ adsorption-desorption analysis. As shown in Figure 3, Fe_3_O_4_@FU NPs have type IV isotherms and type H_3_ hysteresis loops. The BET surface area, average pore diameter and the total pore volume were calculated to be 55.65 m^2^/g, 11 nm, and 1.749 cm^3^/g, respectively.

**Figure 4:**
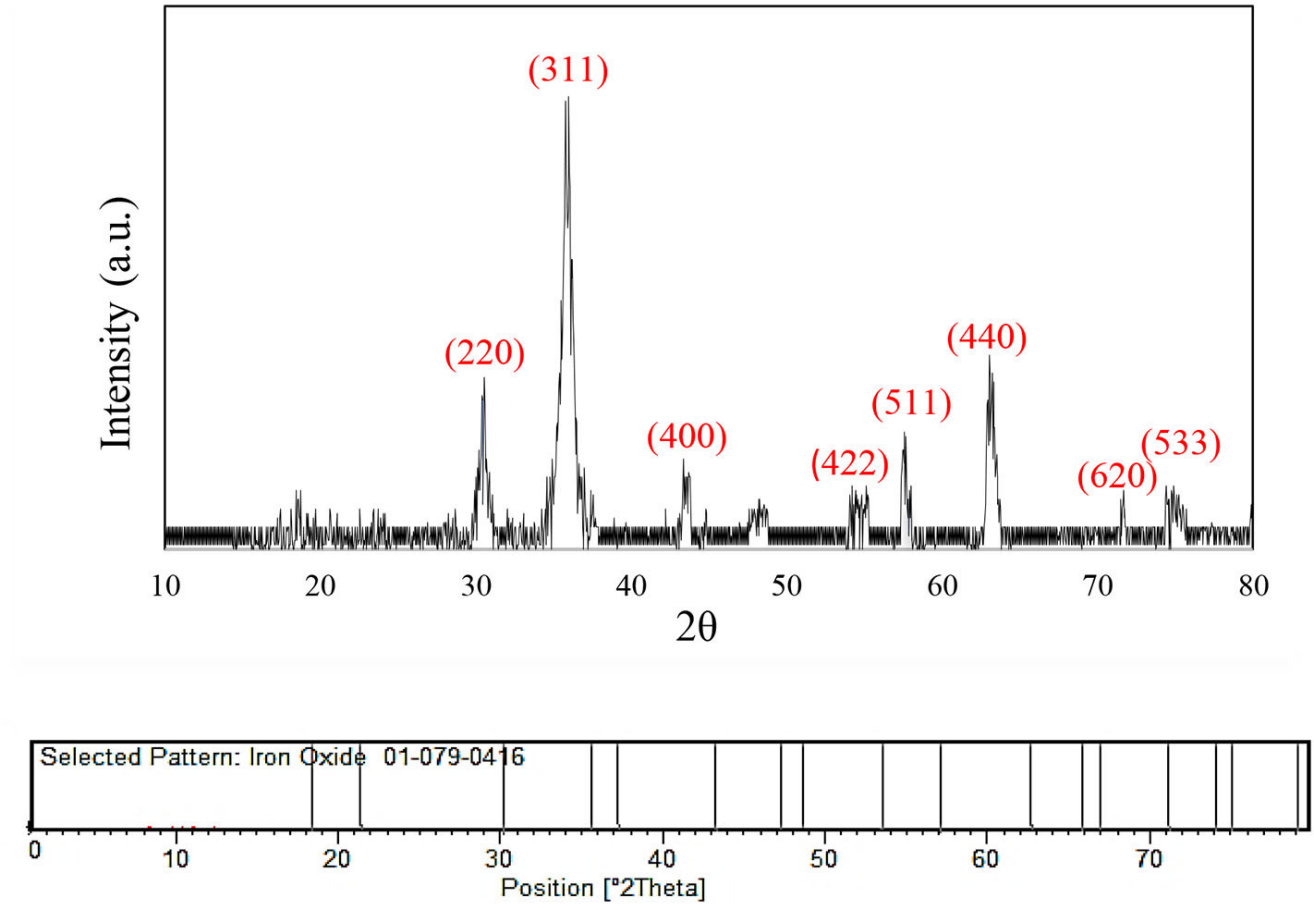
XRD patterns of Fe_3_O_4_@FU NPs.

The XRD patterns of Fe_3_O_4_@FU NPs are presented in Figure 4. As can be seen, diffraction peaks at 2θ values = 30.4°, 35.8°, 43.7°, 55.3°, 57.8°, and 63.3° could be indexed to the presence of all the crystal planes such as (220), (311), (400), (422), (511), and (440) attributed to the cubic inverse spinel structure of Fe_3_O_4_ which matched with the standard pattern (JCPDS-PDF, No. 01-087-2334). These results proved that the crystalline structure of Fe_3_O_4_ was maintained after its decoration with fucoidan polysaccharide.

**Figure 5:**
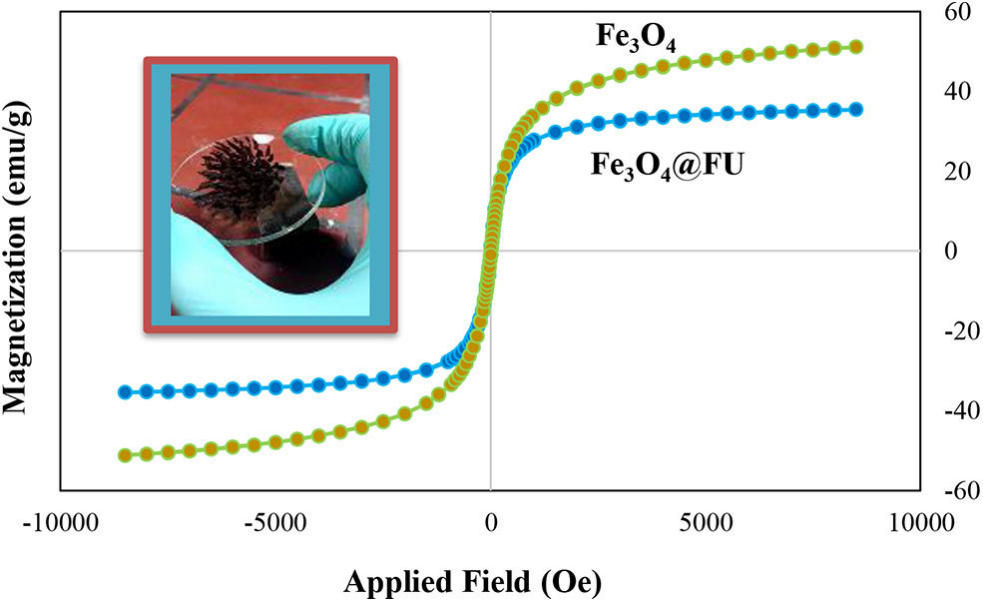
Magnetization curves for Fe_3_O_4_ and the Fe_3_O_4_@FU at room temperature.

The magnetization curves of Fe_3_O_4_ and Fe_3_O_4_@FU NPs measured at room temperature with a vibrating sample magnetometer (VSM) were shown in Figure 5. The hysteresis loops of Fe_3_O_4_@FU NPs showed the superparamagnetic behavior of Fe_3_O_4_@FU NPs. The saturation magnetization (Ms) values for the Fe_3_O_4_ and the Fe_3_O_4_@FU NPs were 51, 35 emu/g, respectively. It is important to note that the saturation magnetization remains sufficient after covering by FU.

**Figure 6:**
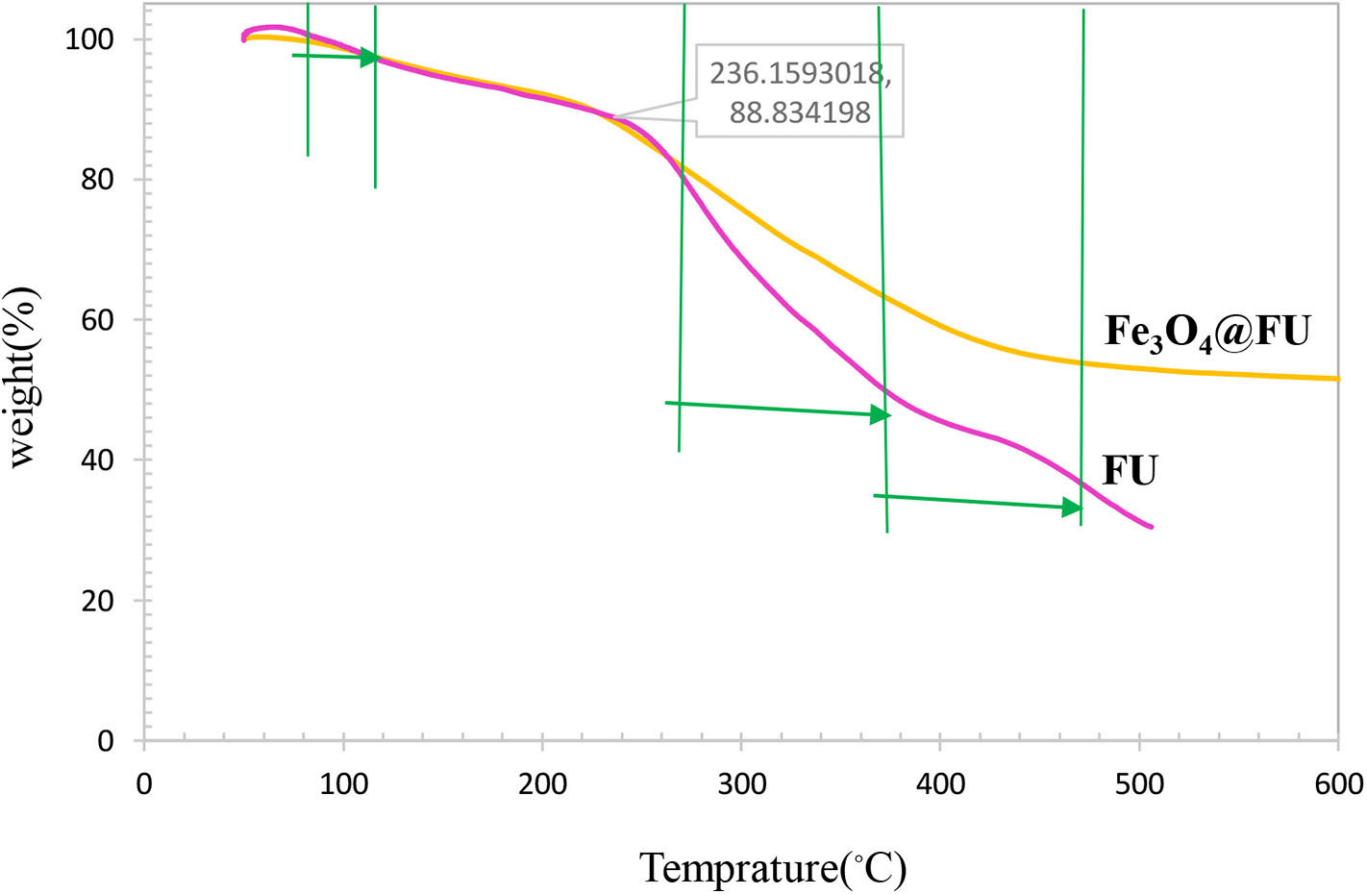
TGA curves of (a) FU (b) Fe_3_O_4_@FU NPs.

The thermogravimetric analysis (TGA) curve of FU and Fe_3_O_4_@FU NPs showed three-stage weight loss in the temperature range from 100 to 500°C (Figure 6). The first weight loss of around 2 wt% ensues at 100°C indicating the evaporation of water or solvent. The next weight loss of about 12 wt% occurs at 240°C and the third weight loss about 42 wt% at 440°C for the decomposition of polysaccharide. Accordingly, the TGA studies showed improved stability for Fe_3_O_4_@FU NPs.

### Catalytic Activity of Magnetic Fe_3_O_4_@FU NPs

The catalytic efficacy of Fe_3_O_4_@FU NPs as a proficient heterogeneous catalyst was investigated in the one-pot reaction between benzil (1 mmol), benzaldehyde (1 mmol), and ammonium acetate (2 mmol) as a model reaction for the synthesis of imidazole derivatives. To determine the role of the catalyst, the model reaction was performed in the absence of the catalyst. The anticipated product was not shown after a long reaction time. The results reveal that the presence of the catalyst has a considerable effect on the formation of these compounds. The model reactions were carried out in the presence of different solvents such as EtOH, H_2_O, THF, and Toluene, CH_3_CN and solvent-free conditions. As the results show, Ethanol (3 ml) was found to be the most effective solvent. To evaluate the optimum catalyst concentration, the model reaction was carried out in the presence of various amounts of catalyst (5, 10, 12, and 15 mg). Consequently, the best yield is accessible in the presence of just 12 mg catalyst, and use of extra amounts of the catalyst (15 mg) did not increase the result to a significant level (Table 1). The model reaction was carried out with FU, Fe_3_O_4_, and Fe_3_O_4_@FU. These results endorsed that Fe_3_O_4_@FU was more suitable for this reaction. Overall, the most significant conditions for the desired products were achieved at reflux under ethanol in the presence of 12 mg magnetic nanocomposite.

**Table 1:** Condition optimization for the synthesis of 2,4,5-trisubstituted imidazole (5k)^a^.

**Entry**	**Catalyst**	**Temp. (^**°**^C)**	**Solvent**	**Catalyst (mg)**	**Time (min)**	**Yield (%)**
1	-	r.t.	Solvent free	-	24 h	-
2	-	Reflux	Ethanol	-	250	Trace
3	FU	Reflux	Ethanol	12	40	70
4	Fe_3_O_4_	Reflux	Ethanol	12	40	55
5	Fe_3_O_4_@FU	Reflux	Ethanol	12	20	96
6	Fe_3_O_4_@FU	Reflux	Ethanol/ Water	12	30	80
7	Fe_3_O_4_@FU	r.t.	Ethanol	12	35	75
8	Fe_3_O_4_@FU	Reflux	Water	12	40	60
9	Fe_3_O_4_@FU	Reflux	CH_3_CN	12	70	48
10	Fe_3_O_4_@FU	Reflux	THF	12	90	40
11	Fe_3_O_4_@FU	Reflux	Toluene	12	60	45
12	Fe_3_O_4_@FU	Reflux	Ethanol	15	20	96
13	Fe_3_O_4_@FU	Reflux	Ethanol	10	30	90
14	Fe_3_O_4_@FU	Reflux	Ethanol	5	50	60

a *Reaction conditions: benzaldehyde (1 mmol), benzil (1 mmol), ammonium acetate (2 mmol), solvent (3 ml)*.

For total assessment of the synthesis of 2,4,5-trisubstituted and 1,2,4,5-tetrasubstituted imidazoles after the mentioned optimized conditions, various aromatic aldehydes and anilines were evaluated which can be seen in Table 2, including both electron-donating and electron-withdrawing substitutions which were studied in these reactions. While the presence of electron-donating groups resulted in the corresponding products being prepared with lower reaction yields, in addition, electron-withdrawing functionalities led to the higher yields with shorter reaction times. Most of the products directly crystallized from the mixture of reaction with high purity with good to excellent yields (80–96%). It should be mentioned that all the products were confirmed by melting points, and some of the products were characterized by NMR spectral data.

**Table 2:** Synthesis of 2,4,5-triaryl **(5a-k)**^a^ and 1,2,4,5- tetraaryl−1*H*-imidazoles **(6l-p)**^b^ catalyzed by Fe_3_O_4_@FU nanocomposite.

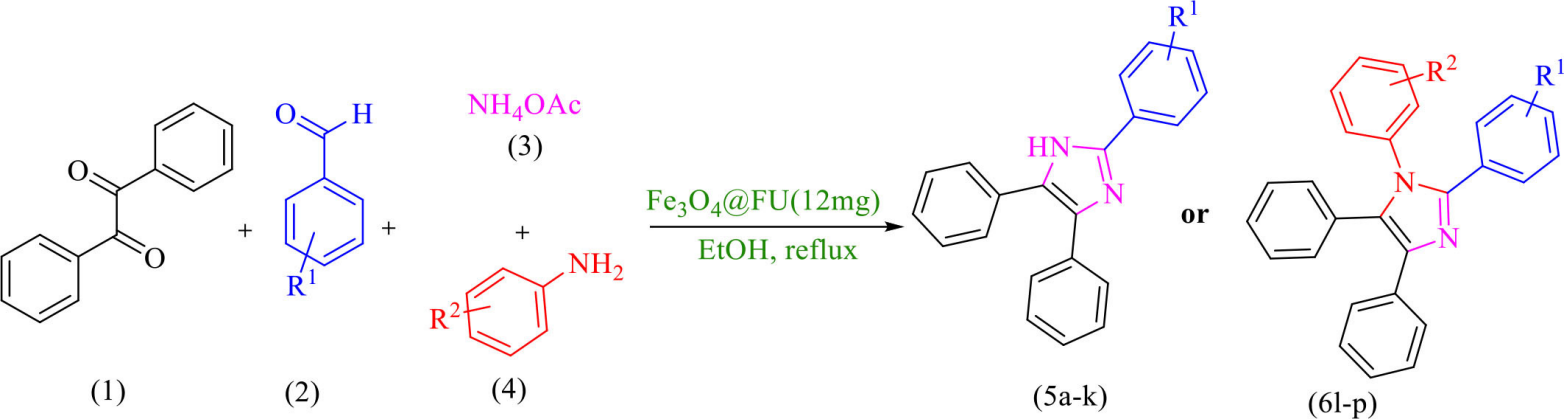						
**Entry**	**R^**1**^-ArCHO**	**R^**2**^-ArNH_**2**_**	**Product^d^**	**Time (min)**	**Yield^c^ (%)**	**M.p (^**°**^C) found/reported**
1	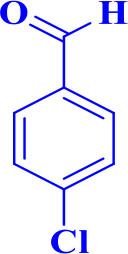	**-**	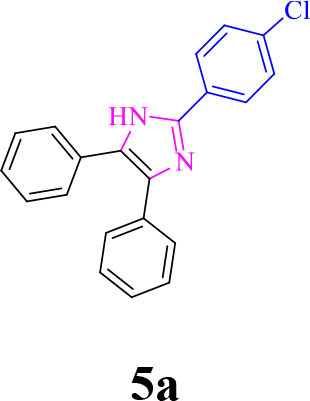	12	96	261–262/260–261 (Salimi et al., 2015)
			**5a**			
2	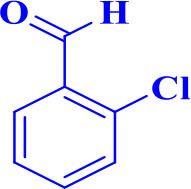	**-**	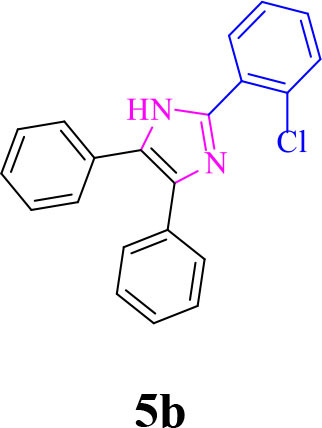	20	90	192–194/191–192 (Shaabani et al., 2017)
			**5b**			
3	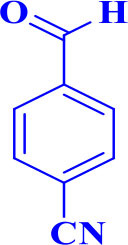	**-**	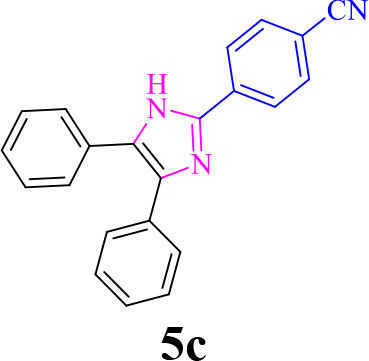	12	96	248–250/248–250 (Marques et al., 2012)
			**5c**			
4	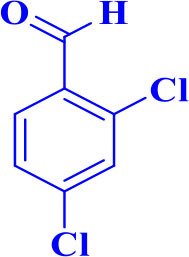	**-**	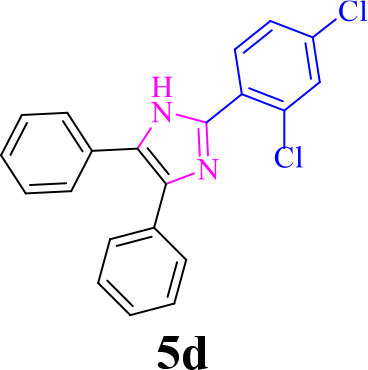	15	91	170–173/171–174 (Amirnejat et al., 2020a)
			**5d**			
5	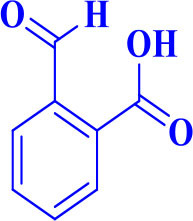	**-**	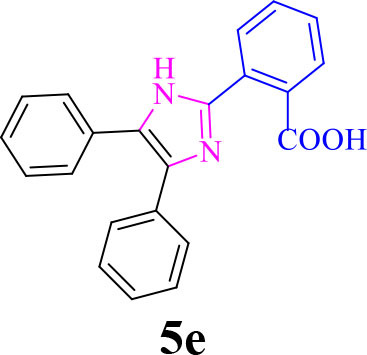	22	90	301–303/300–303 (Amirnejat et al., 2020a)
			**5e**			
6	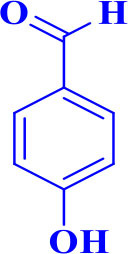	**-**	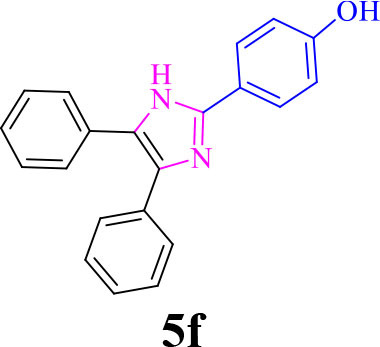	25	88	229–231/230–233 (Momahed Heravi et al., 2019)
			**5f**			
7	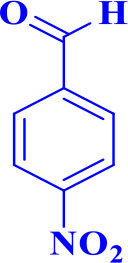	**-**	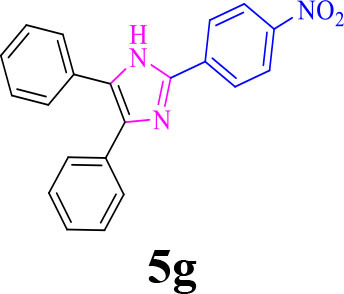	15	95	195–197/196–198 (Shaabani et al., 2016)
			**5g**			
8	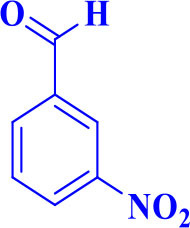	**-**	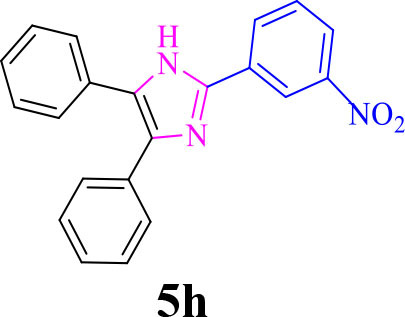	20	86	314–316/313–315 (Momahed Heravi et al., 2019)
			**5h**			
9	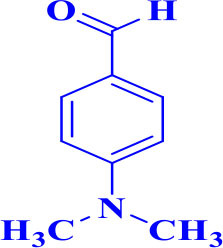	**-**	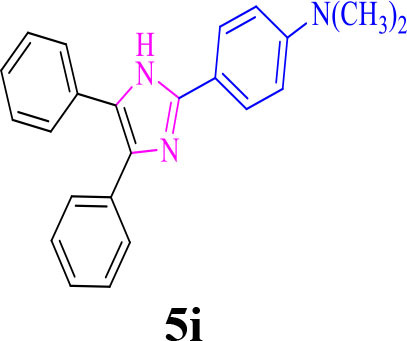	25	80	257–259/256–259 (Salimi et al., 2015)
			**5i**			
10	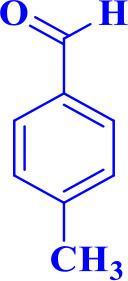	**-**	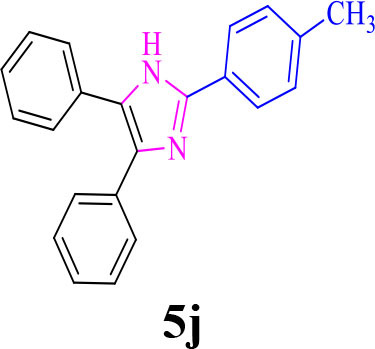	30	80	230–232/230–232 (Mirsafaei et al., 2016)
			**5j**			
11	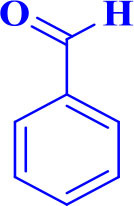	**-**	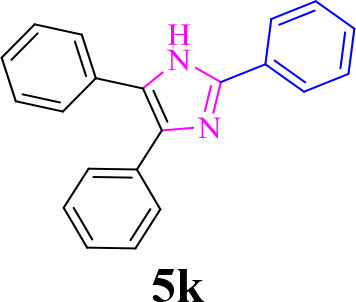	20	90	268–270/270 (Shaterian and Ranjbar, 2011)
			**5k**			
12	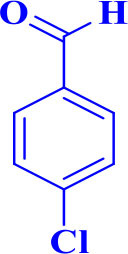	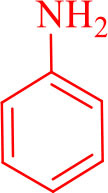	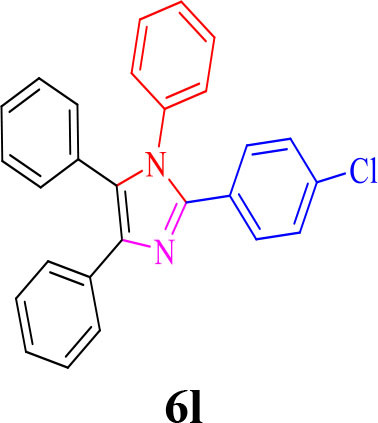	20	94	157–159/156–158 (Salimi et al., 2015)
			**6l**			
13	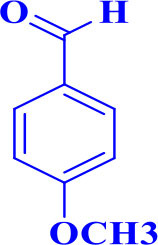	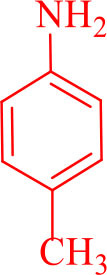	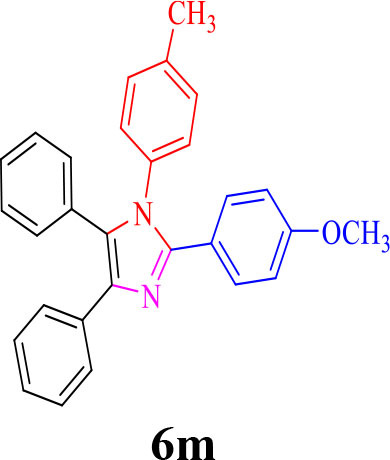	30	85	175–177/176–178 (Ray et al., 2013)
			**6m**			
14	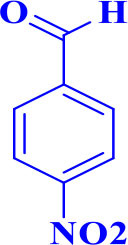	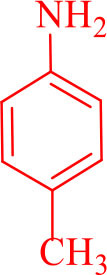	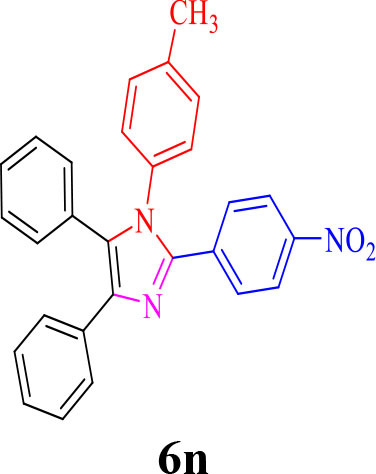	30	90	230–234/232–236 (Salimi et al., 2015)
			**6n**			
15	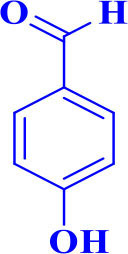	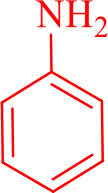	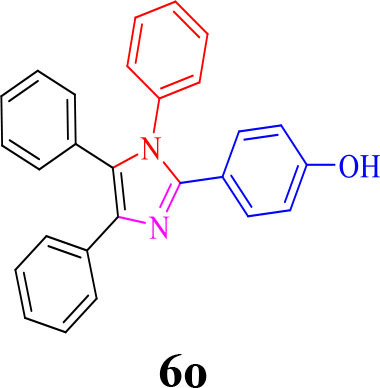	35	83	260–262/257–259 (Mohammadi et al., 2012)
			**6o**			
16	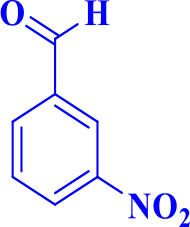	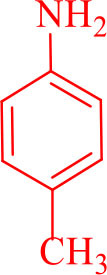	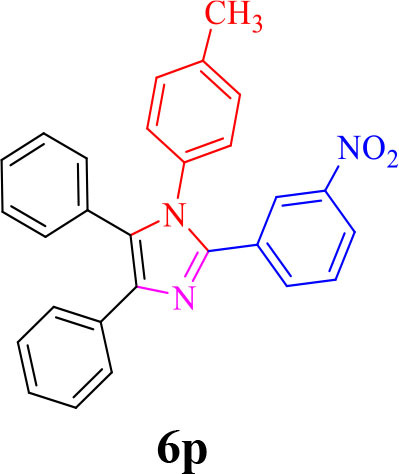	30	87	150–152/150–152 (Salimi et al., 2015)
			**6p**			

a *Reaction conditions: benzil (1 mmol), aldehydes (1 mmol), ammonium acetate (2 mmol), EtOH (3 ml), and Fe_3_O_4_@FU nanocomposite (12 mg) **(5a-k)***.

b *Reaction conditions: benzil (1 mmol), aldehydes (1 mmol), ammonium acetate (2 mmol), aniline (1 mmol), EtOH (3 ml), and Fe_3_O_4_@FU nanocomposite (12 mg) **(6l-p)***.

c *Isolated yields*.

d *The ^1^HNMR spectra for products 5a and 5c have been provided in supplementary file*.

To evaluate the generality of this catalyst in comparison to previously reported results in the literature, Fe_3_O_4_@FU acts as an appropriate green biocatalyst due to the yields of products, reaction time and temperature (Table 3).

**Table 3:** Comparison of the activity of the catalysts in synthesis **5k** with some of the other catalysts reported in the literature.

**Entry**	**Catalyst**	**Reaction condition**	**Reaction time (min)**	**Yield (%)**	**References**
1	[Et_3_NH][HSO_4_](10 mol %)	Solvent-free/	40	89	Deng et al., 2013
		130°C			
2	COPAPSC(20 mg)	Solvent-free/	4h	77	Salimi et al., 2015
		110°C			
3	(CTA)3PMo-MMT nanocomposite (50 mg)	Solvent-free/	60	85	Masteri-Farahani et al., 2020
		100°C			
4	Fe_3_O_4_@FU (12 mg)	EtOH, Reflux	20	90	This work

**Scheme 3:**
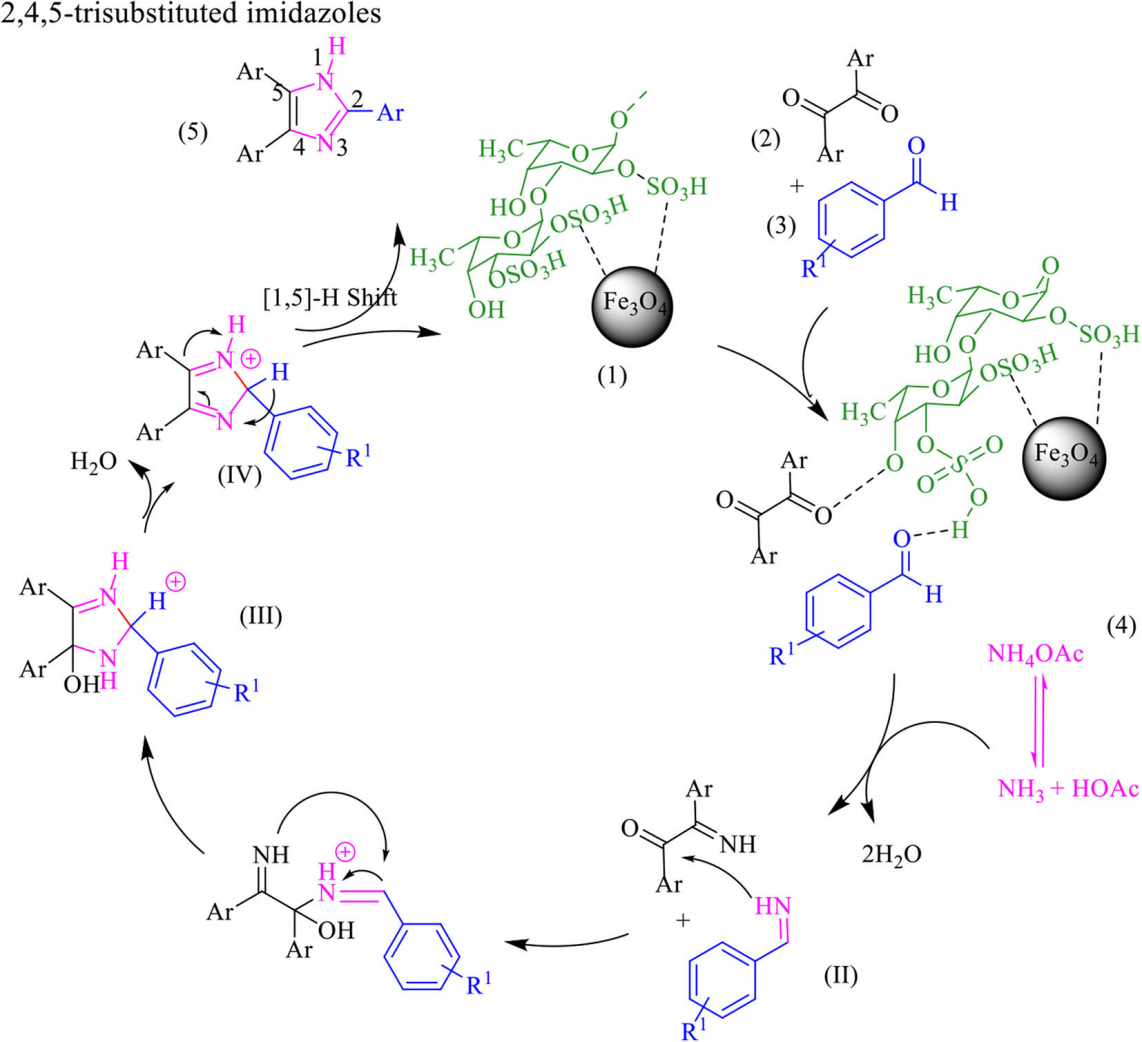
The proposed mechanism for the synthesis of 2,4,5-trisubstituted imidazoles by using Fe_3_O_4_@FU nanocomposite.

The proposed mechanism for the synthesis of tri-substituted imidazoles was shown in Scheme 3. In the first step, the aldehyde and benzil group were activated by the formation of a hydrogen bond with the functional group of fucoidan, followed by the nucleophilic attack of ammonia, coming from the ammonium acetate, the intermediate imine (II) is formed after the removal of H_2_O. Intermediate (II) is then added to benzil forming intermediate (III). Dehydration of intermediate (III) afforded the intermediate (IV), which rearranges via 1,5 H-shift which, followed by deprotonation, gives tri substituted imidazole (5).

**Scheme 4:**
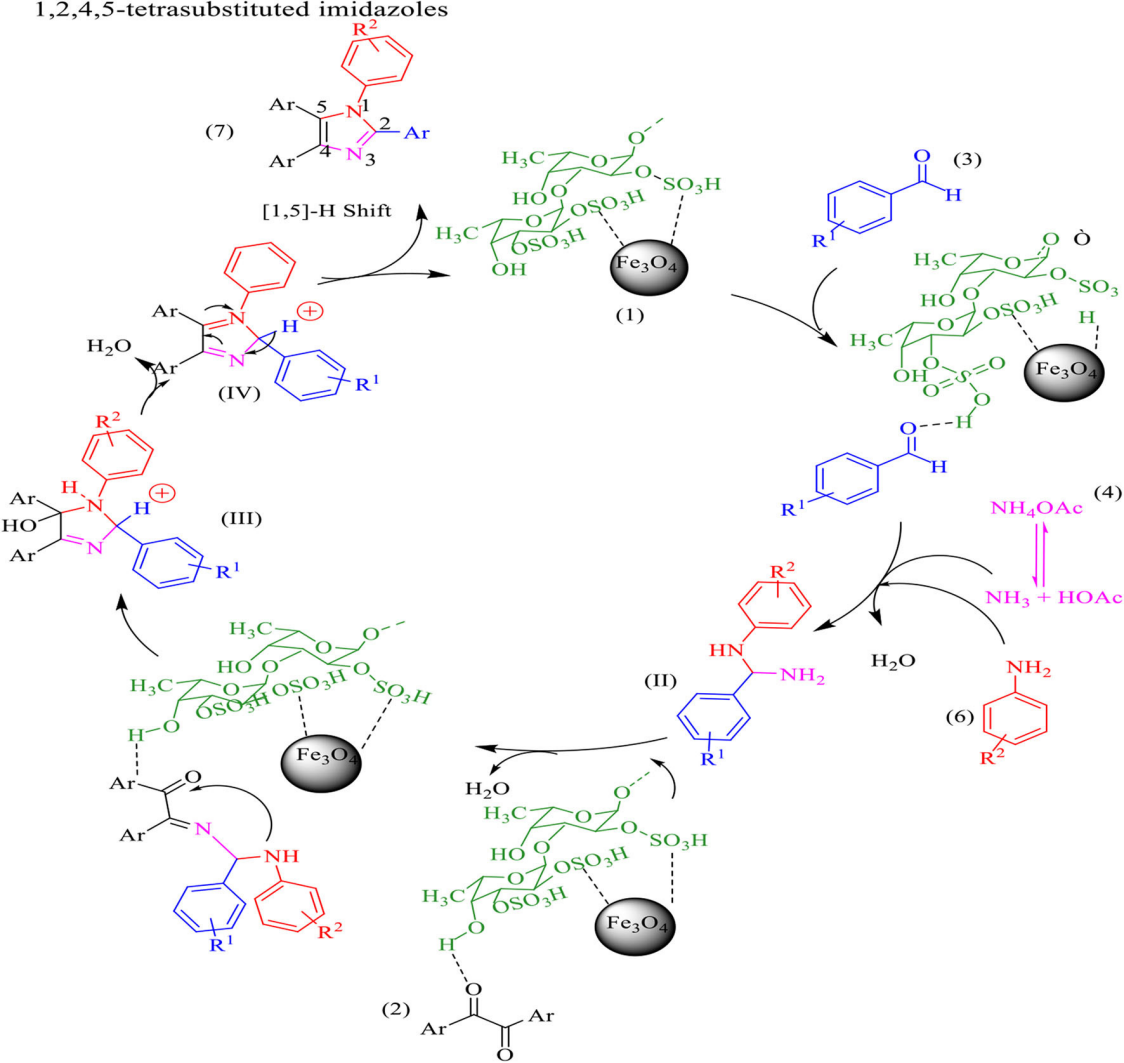
The proposed mechanism for the synthesis of1, 2,4,5-tetra-substituted imidazoles by using Fe_3_O_4_@FU nanocomposite.

The proposed mechanism for the preparation of four one-pot reactions of benzil, aldehyde, and ammonium acetate and amine is shown in Scheme 4. Aldehyde and 1, 2-diketone were first activated by Fe_3_O_4_@FU, then amine was added to the aldehydes forming an imine intermediate which was attacked by ammonia (released from the ammonium acetate) to form the amine intermediate (II). On the other hand, the amine intermediate (II) reacted with the activated carbonyl groups of benzil to form the intermediate (III). Finally, the imidazole derivative was formed after dehydration, followed by a 1,5 H-shift.

**Figure 7:**
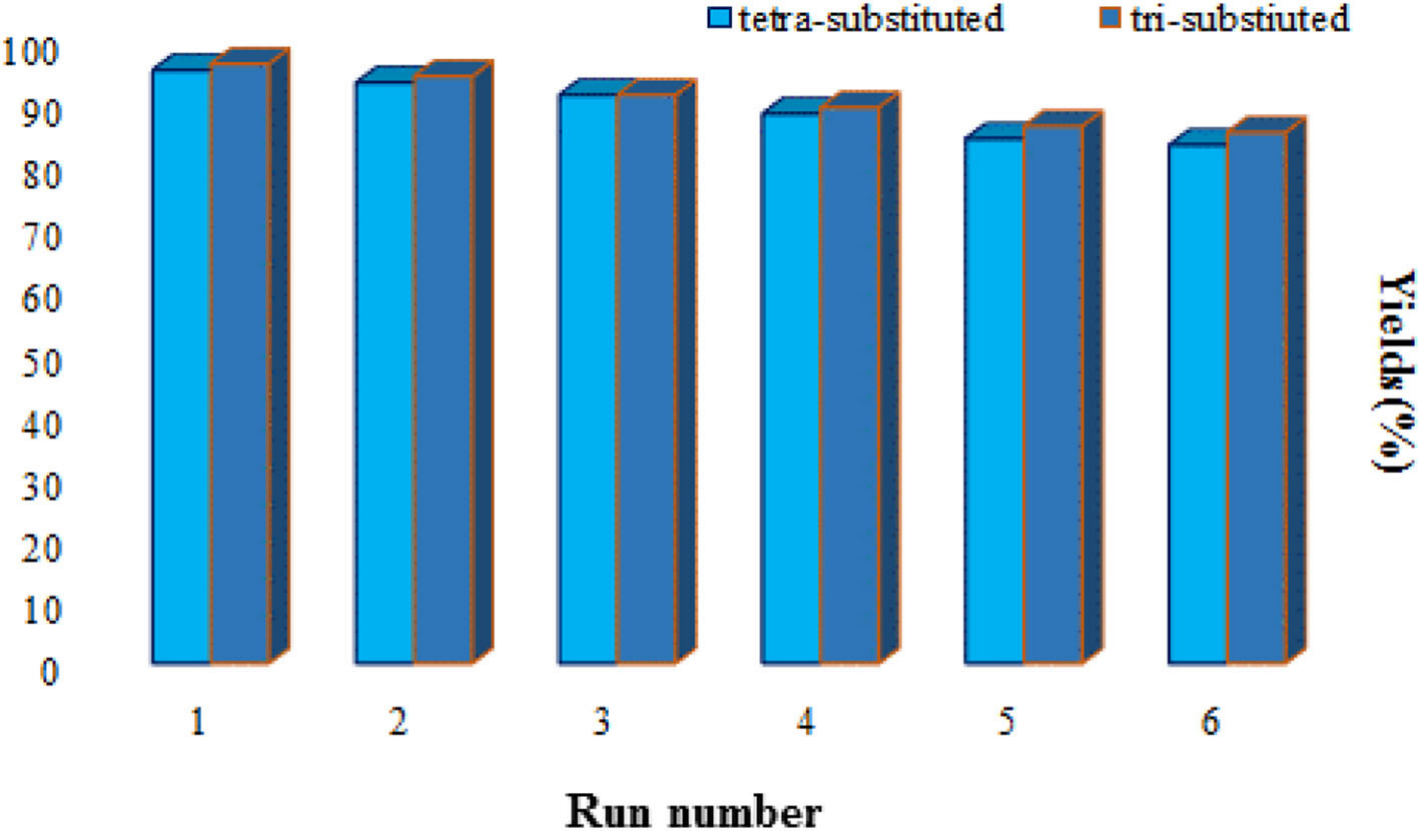
Reusability of Fe_3_O_4_@FU for the synthesis of tri-substituted **(5a)** and tetra-substituted **(6l)** imidazoles.

The main concerns from an economic and environmental aspect, such as recyclability and the ability to reuse the catalyst, were also surveyed. In this regard, after the reaction was completed, the catalyst was collected by an external magnet and then washed with ethyl acetate, n-hexane and ethanol and dried at 50°C in an oven. The recycled catalyst was used six consecutive times in the reaction. According to the results, no appreciable reduction in the efficiency of the catalysts is observed (Figure 7).

**Figure 8:**
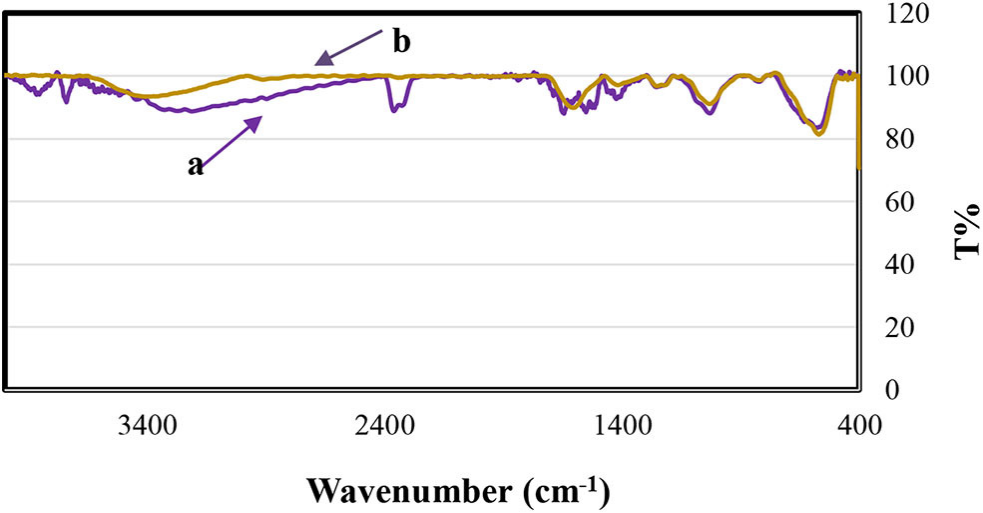
FT-IR spectra of Fe_3_O_4_@FU (a) before being used in the reaction, (b) after being used six times in the reaction.

**Figure 9:**
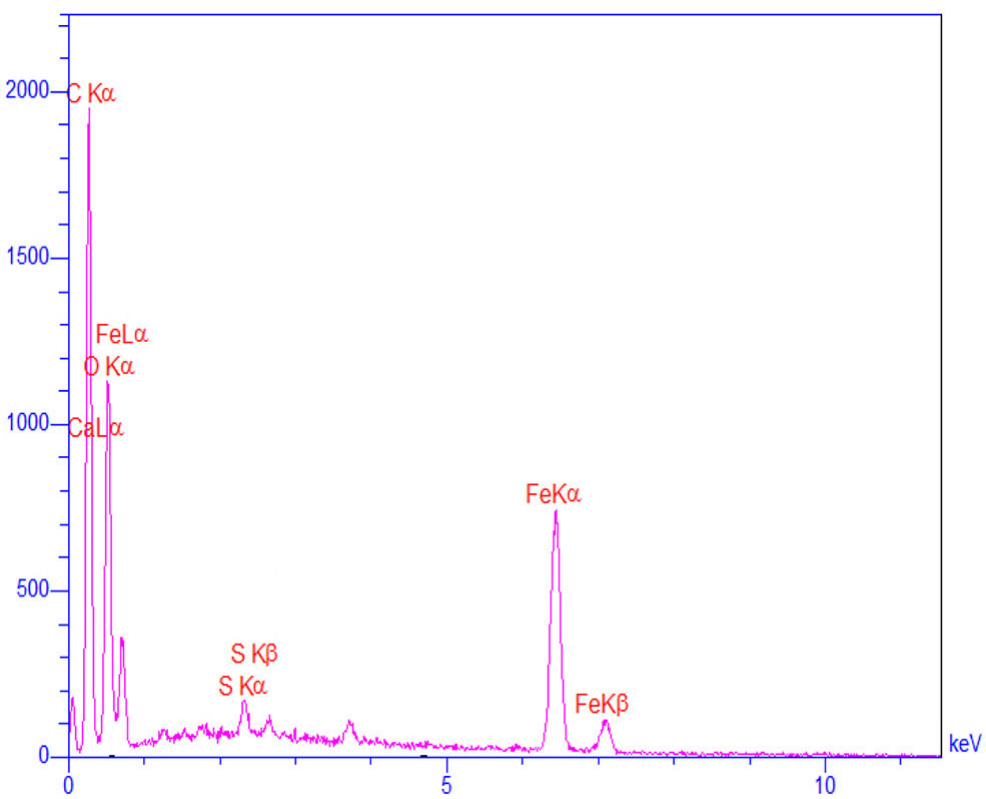
The EDX analysis of recycled Fe_3_O_4_@FU after recycling of Fe_3_O_4_@FU NPs.

The recycled catalyst was identified by EDX and FT-IR analysis. The comparison of the FT-IR spectrum of Fe_3_O_4_@FU before and after six consecutive runs confirms that no definite change in its structure was seen, which can therefore be considered as a recyclable and stable biocatalyst in organic reactions (Figure 8). However, the EDX analysis of the recovered catalyst (Figure 9) showed that a degree of catalyst desulfation and leaching occurred after six runs, which explains the decrease in the yield of reactions.

## Conclusion

In summary, magnetic core-shell structured fucoidan coated Fe_3_O_4_ NPs were *in situ* synthesized and their structural and magnetic properties were investigated. The catalytic property of Fe_3_O_4_@FU was studied in the synthesis of imidazoles derivatives. Outstanding catalytic activity alongside a simple synthesis method, easy processing and separation, a high product yield, and short reaction time make Fe_3_O_4_@FU an attractive bio-based heterogeneous catalyst.

## Data Availability Statement

The raw data supporting the conclusions of this article will be made available by the authors, without undue reservation.

## Author Contributions

MB and SA: investigation and writing—original draft preparation. SJ: project administration, conceptualization, resources, writing—review, and editing. All authors contributed to the article and approved the submitted version.

## Conflict of Interest

The authors declare that the research was conducted in the absence of any commercial or financial relationships that could be construed as a potential conflict of interest.
