# Supplementary material for: Synthesis, Characterization, and Catalytic Properties of Magnetic Fe3O4@FU: A Heterogeneous Nanostructured Mesoporous Bio-Based Catalyst for the Synthesis of Imidazole Derivatives
Source: Front Chem. 2020 Dec 1;8:596029. doi: 10.3389/fchem.2020.596029 (PMC7736414; doi:10.3389/fchem.2020.596029)
Supplement: Supplementary file 1 [file Table_1.DOCX]

Supplementary Material

**^1^HNMR Spectra products 5a and 5c.**


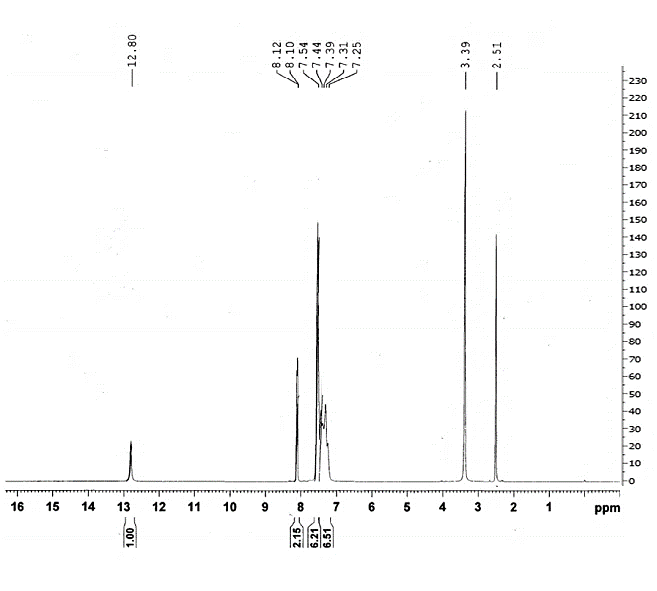

^1^HNMR of 2-(4-Chlorophenyl)-4,5-diphenyl-1*H*-imidazole

**2-(4-Chlorophenyl)-4,5-diphenyl-1*H*-imidazole (5a)**

Color: white; Mp 261–262C; FTIR (KBr, cm^-1^): 3322, 3065, 1596, 1488, 1435, 830; ^1^H NMR (500 MHz, DMSO-d6): δ= 12.80 (s, 1H), 8.11 (d, J = 10Hz, 2H), 7.54–7.25 (m, 12H); Anal. Calcd for C_21_H_15_N_2_Cl: C, 76.25; H, 4.57; N, 8.47.


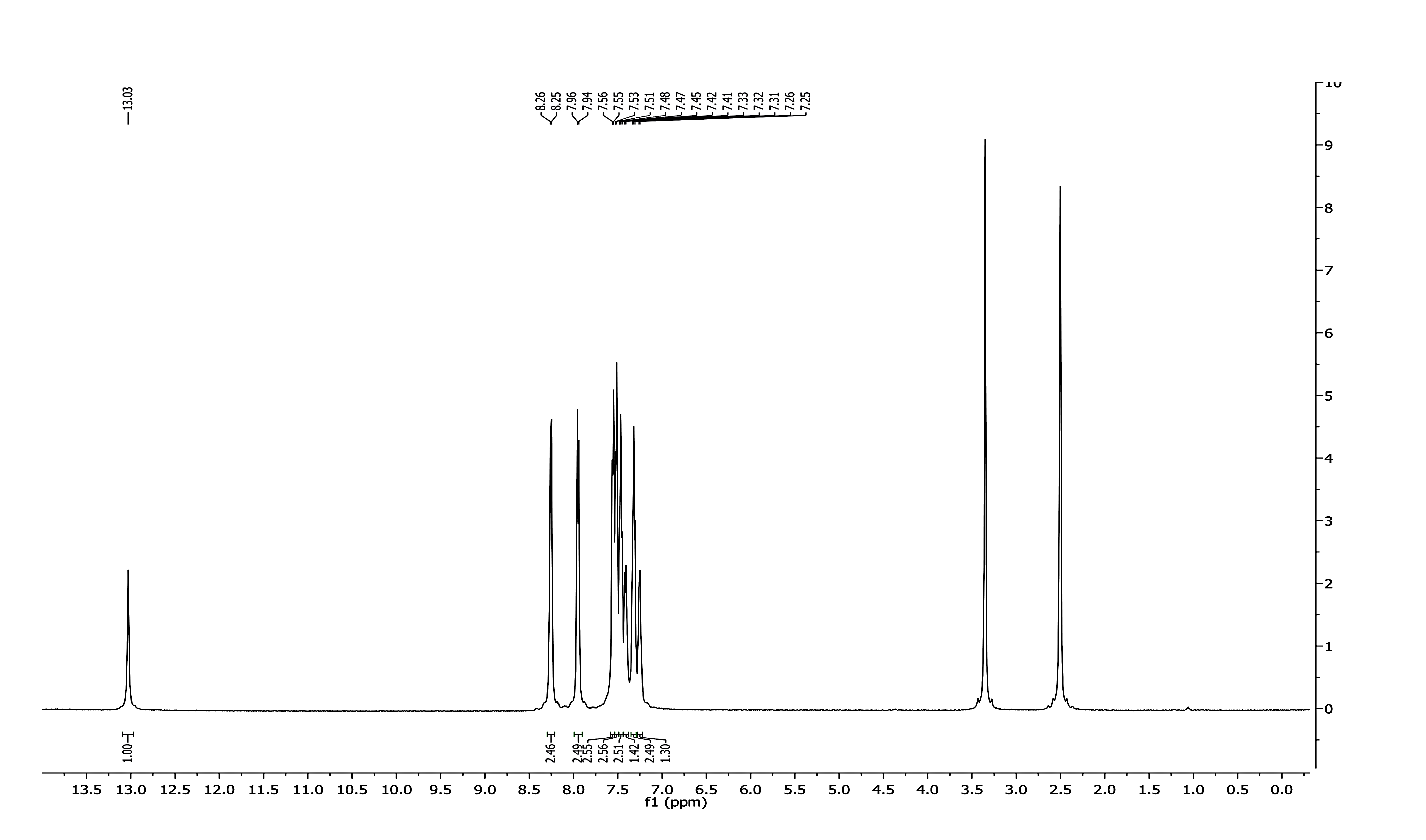

^1^HNMR of 4-(4,5-diphenyl-1*H*-imidazol-2-yl)benzonitrile

**4-(4,5-diphenyl-1*H*-imidazol-2-yl)benzonitrile (5c)**

Color: white; Mp 248–250C; FTIR (KBr, cm^-1^): 3400, 2220, 1601, 1490, 1442, 845; ^1^H NMR (500 MHz, DMSO-d6): δ= 13.03 (s, 1H), 8.25 (d, J = 5Hz, 2H), 7.95 (d, J = 10Hz, 2H), 7.56–7.25 (m, 10H); Anal. Calcd for C_22_H_15_N_3_: C, 82.22; H, 4.70; N, 13.08.
